# Supplementary material for: Constitutive XBP-1s-mediated activation of the endoplasmic reticulum unfolded protein response protects against pathological tau
Source: Nat Commun. 2019 Sep 30;10:4443. doi: 10.1038/s41467-019-12070-3 (PMC6768869; doi:10.1038/s41467-019-12070-3)
Supplement: Supplementary file 1 — Supplementary Information [file 41467_2019_12070_MOESM1_ESM.pdf]

Supplementary Information

Constitutive XBP-1s-mediated activation of the endoplasmic reticulum unfolded protein response protects against pathological tau

Waldherr, et al.

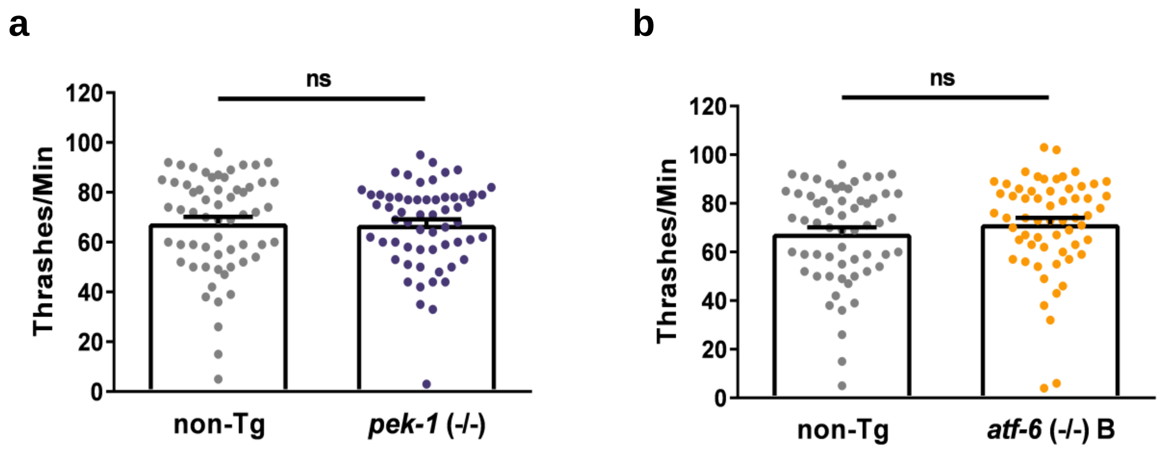

Supplementary Figure 1: Loss of function of the UPR^{ER} PEK-1 and ATF-6 branches does not affect locomotion in *C. elegans*. a Loss of *pek-1* function does not affect behavior observed in a liquid environment [*n* = 60 animals; *N* = 4 biologically independent experiments; statistical analysis is by unpaired *t*-test, two-tailed (ns: *p* = 0.8674)]. **b** Loss of *atf-6* function does not affect behavior observed in a liquid environment. [*n* = 60 animals; *N* = 4 biologically independent experiments; statistical analysis is by unpaired *t*-test, two tailed (ns: *p* = 0.2797)]. Bar graphs represent mean + SEM.

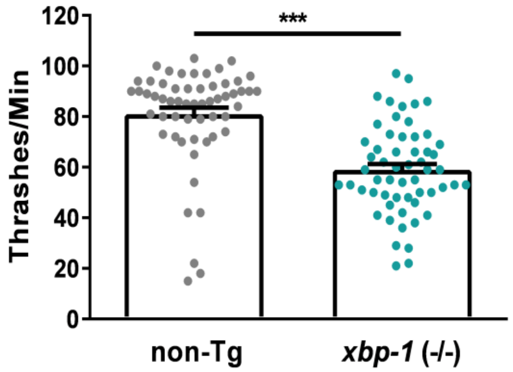

Supplementary Figure 2: Loss of function of the UPR^{ER} XBP-1 branch causes locomotion defects in *C. elegans*. Loss of *xbp-1* function causes mild behavioral defects observed in a liquid environment [n = 57 animals; N = 3 biologically independent experiments; statistical analysis is by unpaired t-test, two-tailed (***$p < 0.0001$)]. Bar graph represents mean + SEM.

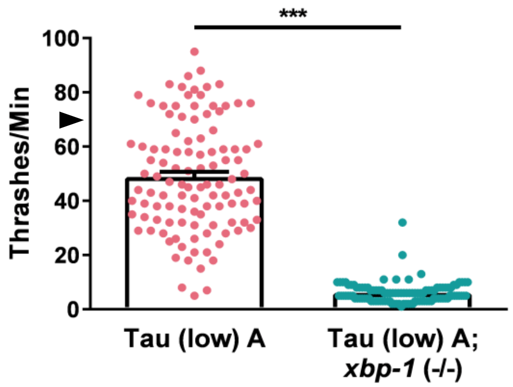

Supplementary Figure 3: Loss of function of the UPR^{ER} XBP-1 branch enhances locomotion defects in an independent tau transgenic *C. elegans* model. Loss of *xbp-1* function in a wildtype tau low expression background enhances mild behavioral defects observed in a liquid environment [n = 110 animals; N = 7 biologically independent experiments; statistical analysis is by unpaired t-test, two tailed (***p < 0.0001)]. Bar graph represents mean + SEM. Arrowhead on *y*-axis denotes non-Tg animals average ~70 thrashes/minute under standard laboratory conditions.

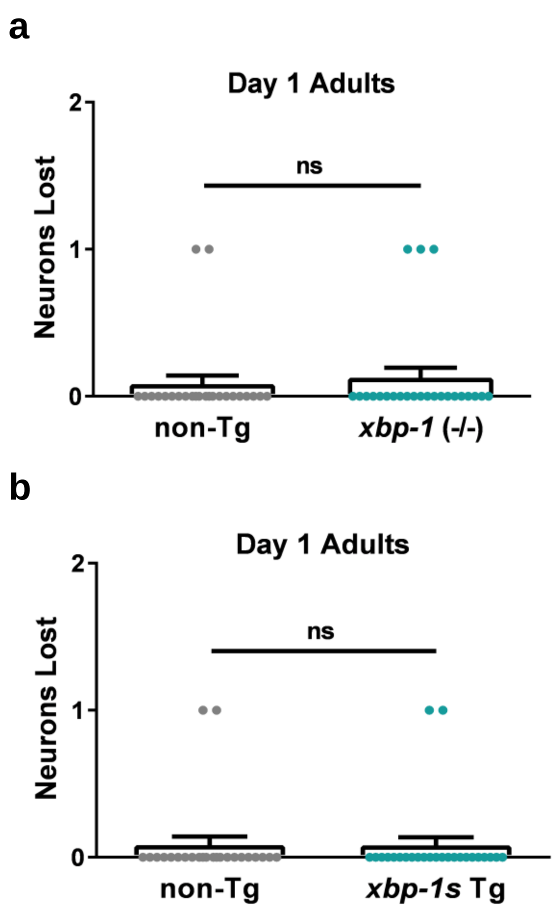

Supplementary Figure 4: Genetic manipulation of the UPR^{ER} XBP-1 branch does not cause neuronal loss in *C. elegans*. a Loss of *xbp-1* function does not cause neuronal loss in adult animals. The number of D-type GABAergic ventral nerve cord neurons lost at day one of adulthood [*n* = 24 animals; *N* = 2 biologically independent experiments; statistical analysis is by unpaired *t*-test, two-tailed (ns: *p* = 0.6451)] is plotted. **b** Neuronal overexpression of *xbp-1s* does not cause neuronal loss in adult animals. The number of D-type GABAergic ventral nerve cord neurons lost at day one of adulthood [*n* = 24 and 25 animals, respectively; *N* = 2 biologically independent experiments; statistical analysis is by unpaired *t*-test, two-tailed (ns: *p* = 0.9669)] is plotted. Bar graph represents mean + SEM.

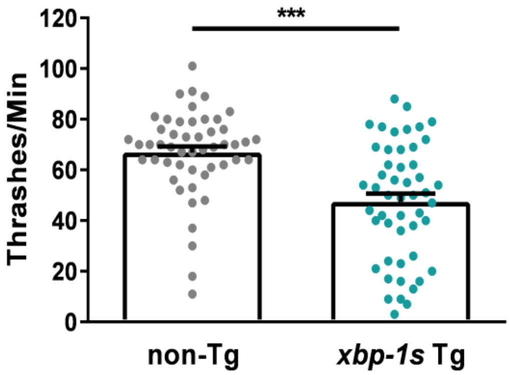

Supplementary Figure 5: Constitutive UPR^{ER} activation by neuronal overexpression of *xbp-1s* causes behavioral defects in *C. elegans*. Neuronal overexpression of *xbp-1*s causes mild behavioral defects observed in a liquid environment [*n* = 50 animals; *N* = 3 biologically independent experiments; statistical analysis is by unpaired *t*-test, two-tailed (***$p < 0.0001$)]. Bar graph represents mean + SEM.

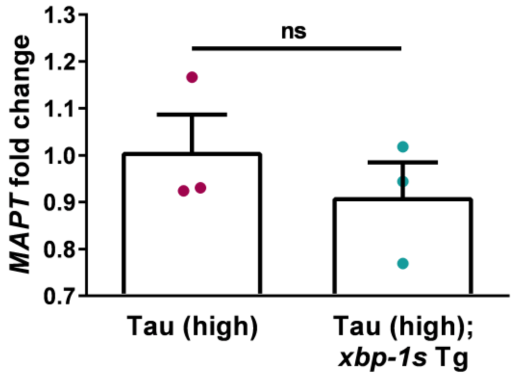

Supplementary Figure 6: Constitutive UPR^{ER} activation by neuronal overexpression of *xbp-1s* does not alter human *MAPT* transcript levels in tau transgenic *C. elegans*. Neuronal overexpression of *xbp-1*s in a wildtype tau high expression background does not affect human *MAPT* gene expression levels in adult animals. *MAPT* gene expression levels normalized to *C. elegans rpl-32* gene expression levels are plotted [N = 3 biologically independent experiments (with three technical replicates within each experiment); statistical analysis is by unpaired t-test, two-tailed (ns: p = 0.4250)]. Bar graph represents mean + SEM.

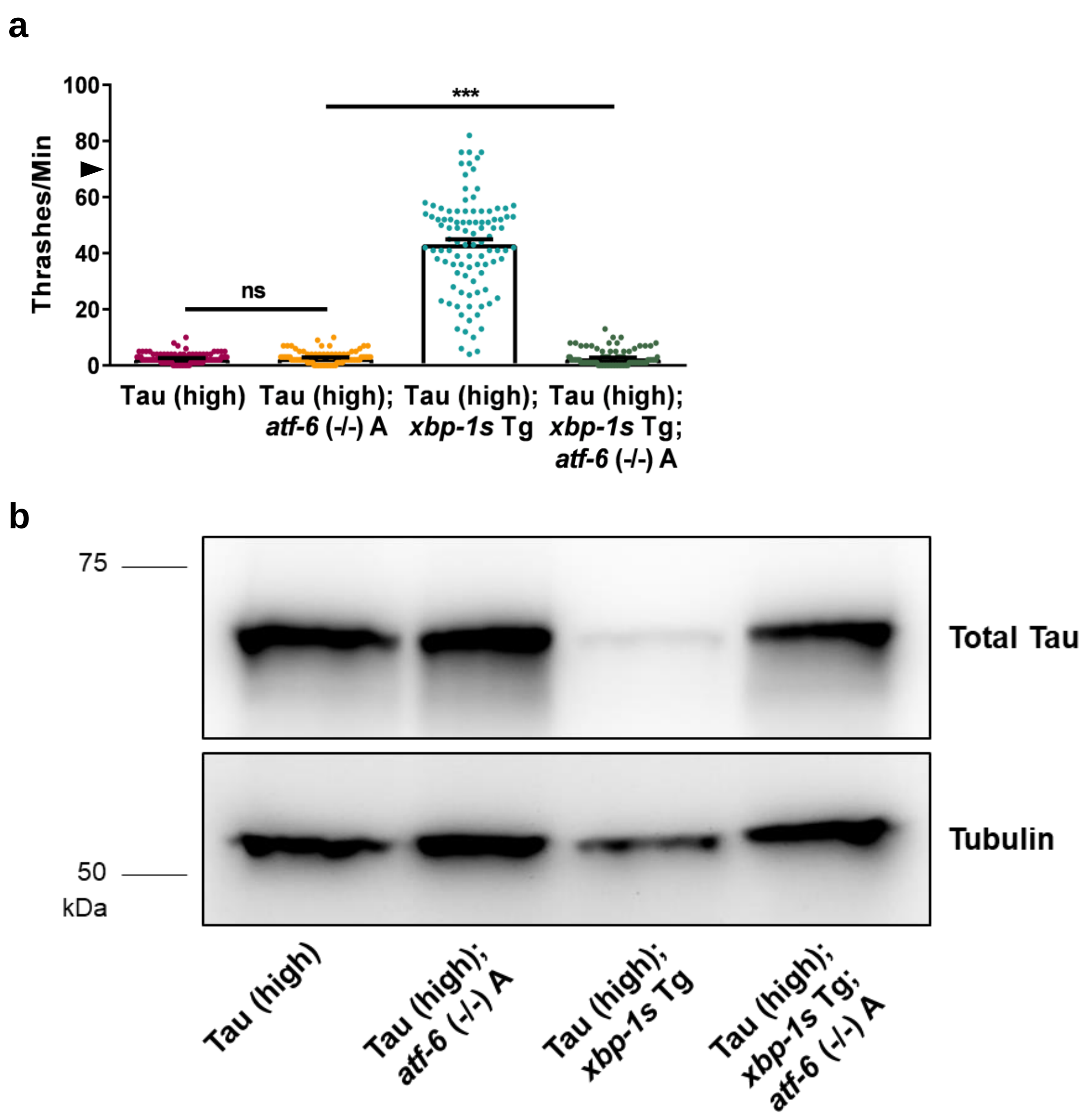

Supplementary Figure 7: Loss of function of the UPR^{ER} ATF-6 branch using an independent allele abolishes *xbp-1s*-mediated tauopathy suppression in tau transgenic *C. elegans*. a Loss of *atf-6* function abolishes the ability of neuronal overexpression of *xbp-1s* in a wildtype tau high expression background to suppress severe behavioral defects observed in a liquid environment [n = 100 animals; N = 7 biologically independent experiments; statistical analysis is by one-way ANOVA, followed by Tukey's post-test (***$p < 0.0001$)]. Bar graph represents mean + SEM. Arrowhead on *y*-axis denotes non-Tg animals average ~70 thrashes/minute under standard laboratory conditions. **b** Loss of *atf-6* function abolishes the ability of neuronal overexpression of *xbp-1s* in Tau (high) animals to decrease soluble tau protein levels. Immunoblots for total tau and tubulin are shown for one biologically independent experiment.

Supplementary Table 1: *C. elegans* Strains and Transgenics

**Abbreviation**	**Strain #**	**Genotype**	**Outcrossed**	**Source**
non-Tg	N2	Bristol, Great Britain wildtype isolate	0x	CGC
Tau (WT High)	CK144	*aex-3p::*hTau (4R1N); *myo-2p::gfp*	2x	[20]
Tau (WT Low) A	CK1044	*aex-3p::*hTau (4R1N); *myo-2p::gfp*	2x	[20]
Tau (WT Low) B	CK1441	*aex-3p::*hTau (4R1N); *myo-2p::dsRED*	2x	This Study
xbp-1s Tg	AGD927	uthls270 [*rab-3p::xbp-1s; myo-2p::tdTomato*]	8x	[12]
GABAergic Reporter	EG1285	*lin-15B* & *lin-15A(n765)*; *oxIs12* [*unc-47p::gfp*] *X*	0x	CGC
pek-1 (-/-)	RB545	*pek-1(ok275) X*	2x	CGC
atf-6 (-/-) A	Tm1153	*atf-6(tm1153) X*	2x	Crowder lab
atf-6 (-/-) B	RB772	*atf-6(ok551) X*	2x	CGC
xbp-1 (-/-)	SJ17	*xbp-1(zc12) III*; *zcls4* [*hsp-4p::gfp*] *V*	2x	CGC
sel-11 (-/-)	MT14875	*nDf59 V*	2x	CGC
CP450 (ER)	CX10344	*unc-25::calf-1::gfp, unc-25::CP450::mCherry, odr-1::dsRED*	0x	[24]

Supplementary Table 2: Protein Antibodies

Antigen	Clone/ Product Name	Dilution	Host Species	Source	Catalog #
β-Tubulin	E7 mAb	1:5,000	Mouse	Developmental Studies Hybridoma Bank (Iowa City, IA, USA)	N/A
Tau (Total)	SP70 pAb	1:1,000	Rabbit	Rockland Immunochemicals Inc. (Limerick, PA, USA)	200-C01-B33
pTau Ser202	CP13 mAb	1:500	Mouse	Peter Davies (Litwin-Zucker Research Center for the Study of Alzheimer's Disease, The Feinstein Institute of Medical Research, Northwell Health, Manhasset, NY, USA)	N/A
pTau Ser396/Ser404	PHF-1 mAb	1:2,000	Mouse		
pTau Ser422	EPR2866 mAb	1:500	Rabbit	Abcam (Cambridge, UK)	ab79415
pTau Thr181	AT270 mAb	1:15,000	Mouse	ThermoFisher Scientific (Waltham, MA, USA)	MN1050
Tau (Total)	K9JA (DAKO) pAb	1:250	Rabbit	Agilent Technologies, Inc. (Santa Clara, CA, USA)	A002401-2
mCherry	2F4 mAb	1:250	Mouse	Elabscience Biotechnology, Inc. (Houston, TX, USA)	E-AB-20087
2^0 Ab Mouse	Horseradish Peroxidase α-Ms IgG (H+L)	1:5,000	Goat	Jackson Immunoresearch (West Grove, PA, USA)	115-035-146
2^0 Ab Rabbit	Horseradish Peroxidase α-Rb IgG (H+L)	1:5,000	Goat	Jackson Immunoresearch (West Grove, PA, USA)	111-035-144
2^0 Ab Mouse	Alexa Fluor® 568 Goat α-Ms IgG (H+L)	1:1,000	Goat	Invitrogen (Carlsbad, CA, USA)	A-11004
2^0 Ab Rabbit	Alexa Fluor® 647 Goat α-Rb IgG (H+L)	1:1,000	Goat	Invitrogen (Carlsbad, CA, USA)	A-21245

Supplementary Table 3: Measurement of pTau Immunoblot Signal

	Normalized to Tubulin			Normalized to Total Tau		
Protein	Tau (high) Mean +/- SEM (a.u.)	Tau (high); *xbp-1s* Mean +/- SEM (a.u.)	*p*-value	Tau (high) Mean +/- SEM (a.u.)	Tau (high); *xbp-1s* Mean +/- SEM (a.u.)	*p*-value
pTau Thr181	0.81 +/- 0.040	0.079 +/- 0.021	*** (0.0006)	0.69 +/- 0.082	0.40 +/- 0.055	* (0.0254)
pTau Ser202	0.73 +/- 0.057	0.058 +/- 0.0040	** (0.0012)	0.63 +/- 0.090	0.34 +/- 0.055	ns (0.1051)
pTau Ser396/Ser 404	0.57 +/- 0.17	0.059 +/- 0.028	* (0.0386)	0.52 +/- 0.18	0.27 +/- 0.12	* (0.0412)
pTau Ser422	0.57 +/- 0.11	0.049 +/- 0.020	* (0.0135)	0.50 +/- 0.12	0.24 +/- 0.090	* (0.0478)
Total Tau	1.21 +/- 0.14	0.18 +/- 0.042	** (0.0061)			

Densitometry analysis of chemiluminescence signals for phosphorylated tau species normalized to tubulin and total tau (*N* = 4 biologically independent experiments; statistical analysis is by paired *t*-test, two-tailed; a.u. = arbitrary units).
