# Supplementary material for: Constitutive XBP-1s-mediated activation of the endoplasmic reticulum unfolded protein response protects against pathological tau
Source: Nat Commun. 2019 Sep 30;10:4443. doi: 10.1038/s41467-019-12070-3 (PMC6768869; doi:10.1038/s41467-019-12070-3)
Supplement: Supplementary file 3 — Reporting Summary [file 41467_2019_12070_MOESM3_ESM.pdf]

nature research

Corresponding author(s): Brian Kraemer

Last updated by author(s): Jul 19, 2019

Reporting Summary

Nature Research wishes to improve the reproducibility of the work that we publish. This form provides structure for consistency and transparency in reporting. For further information on Nature Research policies, see Authors & Referees and the Editorial Policy Checklist.

Statistics

For all statistical analyses, confirm that the following items are present in the figure legend, table legend, main text, or Methods section.

n/a	Confirmed	
☐	☒	The exact sample size (n) for each experimental group/condition, given as a discrete number and unit of measurement
☐	☒	A statement on whether measurements were taken from distinct samples or whether the same sample was measured repeatedly
☐	☒	The statistical test(s) used AND whether they are one- or two-sided<br>*Only common tests should be described solely by name; describe more complex techniques in the Methods section.*
☒	☐	A description of all covariates tested
☐	☒	A description of any assumptions or corrections, such as tests of normality and adjustment for multiple comparisons
☐	☒	A full description of the statistical parameters including central tendency (e.g. means) or other basic estimates (e.g. regression coefficient) AND variation (e.g. standard deviation) or associated estimates of uncertainty (e.g. confidence intervals)
☒	☐	For null hypothesis testing, the test statistic (e.g. F, t, r) with confidence intervals, effect sizes, degrees of freedom and P value noted<br>*Give P values as exact values whenever suitable.*
☒	☐	For Bayesian analysis, information on the choice of priors and Markov chain Monte Carlo settings
☒	☐	For hierarchical and complex designs, identification of the appropriate level for tests and full reporting of outcomes
☒	☐	Estimates of effect sizes (e.g. Cohen's d, Pearson's r), indicating how they were calculated

Our web collection on statistics for biologists contains articles on many of the points above.

Software and code

Policy information about availability of computer code

Data collection	UVP chemiluminescence acquisition software was used to capture images and ImageJ was used for signal quantitation.
Data analysis	Statistical analysis performed with GraphPad Software

For manuscripts utilizing custom algorithms or software that are central to the research but not yet described in published literature, software must be made available to editors/reviewers. We strongly encourage code deposition in a community repository (e.g. GitHub). See the Nature Research guidelines for submitting code & software for further information.

Data

Policy information about availability of data

All manuscripts must include a data availability statement. This statement should provide the following information, where applicable:

- Accession codes, unique identifiers, or web links for publicly available datasets
- A list of figures that have associated raw data
- A description of any restrictions on data availability

Data relevant to this study is available from the authors upon reasonable request.

Field-specific reporting

Please select the one below that is the best fit for your research. If you are not sure, read the appropriate sections before making your selection.

☒ Life sciences ☐ Behavioural & social sciences ☐ Ecological, evolutionary & environmental sciences

For a reference copy of the document with all sections, see nature.com/documents/nr-reporting-summary-flat.pdf

Life sciences study design

All studies must disclose on these points even when the disclosure is negative.

Sample size	Sample size was chosen on the basis of experimental feasibility. For each experiment each group was greater than 24 animals. (n=24 for neurodegeneration assays, n= 100 for behavioral analysis, population size ~10,000 for immunoblotting experiments).
Data exclusions	none
Replication	In all cases experiments were conducted in at least triplicate. When additional alleles and tau transgenic strains were available, key results were confirmed using a second mutant allele and tau transgenic strain.
Randomization	Individual animals were selected randomly from culture plates by use of a wire pick. Expression of approriate transgene markers was confirmed always by observation prior to the experiment.
Blinding	Where feasible behavioral analysis the experimenter was blinded to animal genotype.

Reporting for specific materials, systems and methods

We require information from authors about some types of materials, experimental systems and methods used in many studies. Here, indicate whether each material, system or method listed is relevant to your study. If you are not sure if a list item applies to your research, read the appropriate section before selecting a response.

Materials & experimental systems

n/a	Involved in the study	
☐	☒	Antibodies
☒	☐	Eukaryotic cell lines
☒	☐	Palaeontology
☐	☒	Animals and other organisms
☒	☐	Human research participants
☒	☐	Clinical data

Methods

n/a	Involved in the study	
☒	☐	ChIP-seq
☒	☐	Flow cytometry
☒	☐	MRI-based neuroimaging

Antibodies

Antibodies used	See Table S1 for all antibody details.
Validation	All antibodies were validated against non-Tg animals for human tau; These antibodies are also routinely validated against tau Tg mouse brains and tau KO mouse brains. For other proteins detected by immunoblot N2 was compared to non-Tg and KO animals.

Animals and other organisms

Policy information about studies involving animals; ARRIVE guidelines recommended for reporting animal research

Laboratory animals	C. elegans, background strain N2, Hermaphrodite, young adult, day 4 adult
Wild animals	none
Field-collected samples	none
Ethics oversight	VA PSHCS R&D Committee, Biosafety Subcommittee

Note that full information on the approval of the study protocol must also be provided in the manuscript.
