# Supplementary material for: Constitutive XBP-1s-mediated activation of the endoplasmic reticulum unfolded protein response protects against pathological tau
Source: Nat Commun. 2019 Sep 30;10:4443. doi: 10.1038/s41467-019-12070-3 (PMC6768869; doi:10.1038/s41467-019-12070-3)
Supplement: Supplementary file 4 — Source Data [file 41467_2019_12070_MOESM4_ESM.zip › Source Data - Immunoblots.pdf]

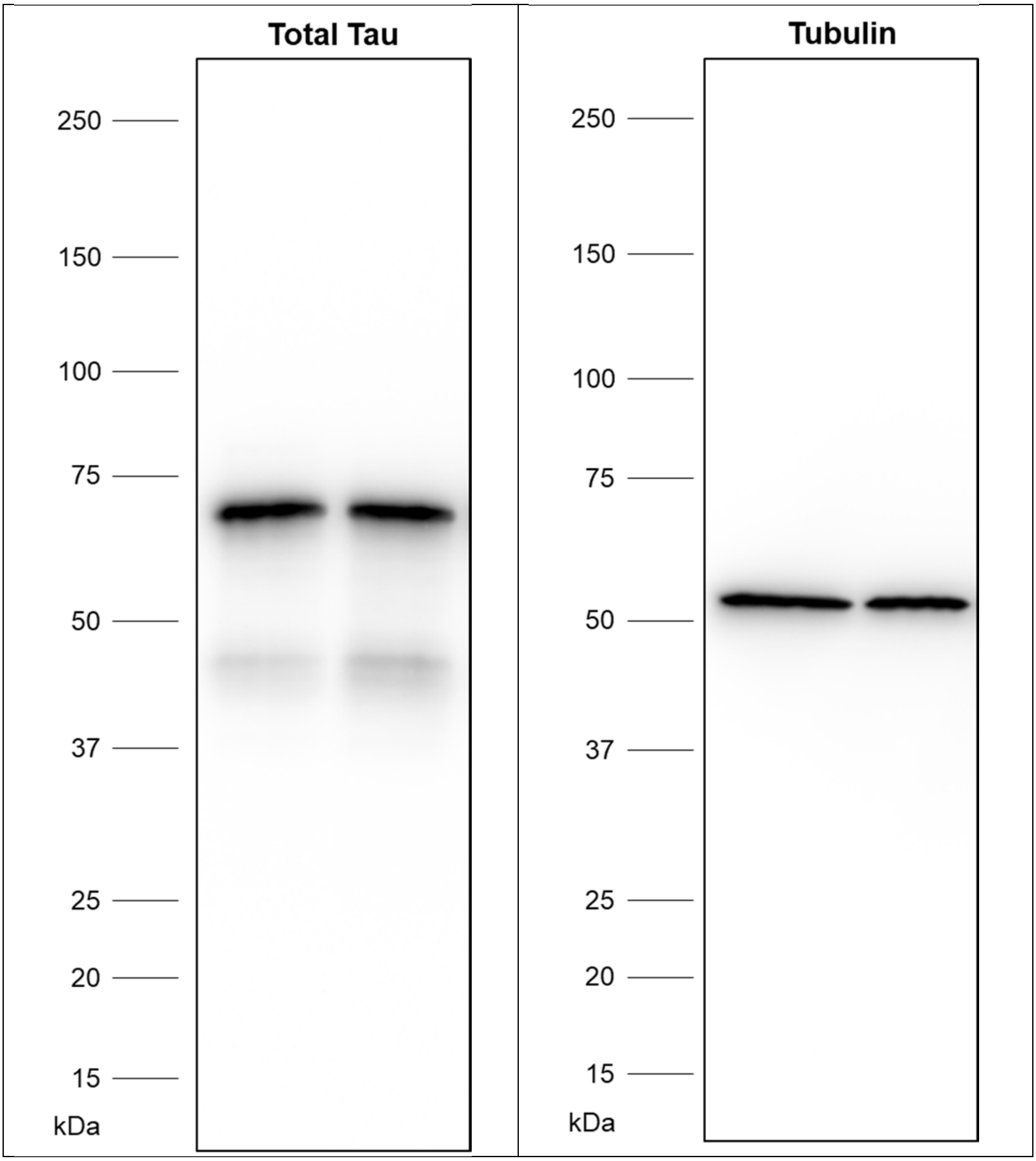

Figure 1d: *atf-6* loss of function in Tau (low) A background does not affect soluble tau protein levels. Representative full-length immunoblots of total tau and tubulin are shown for one of three independent biological replicates for each genotype. Loading order from left to right in each blot is Tau (low) A, Tau (low) A; *atf-6* (-/-) A.

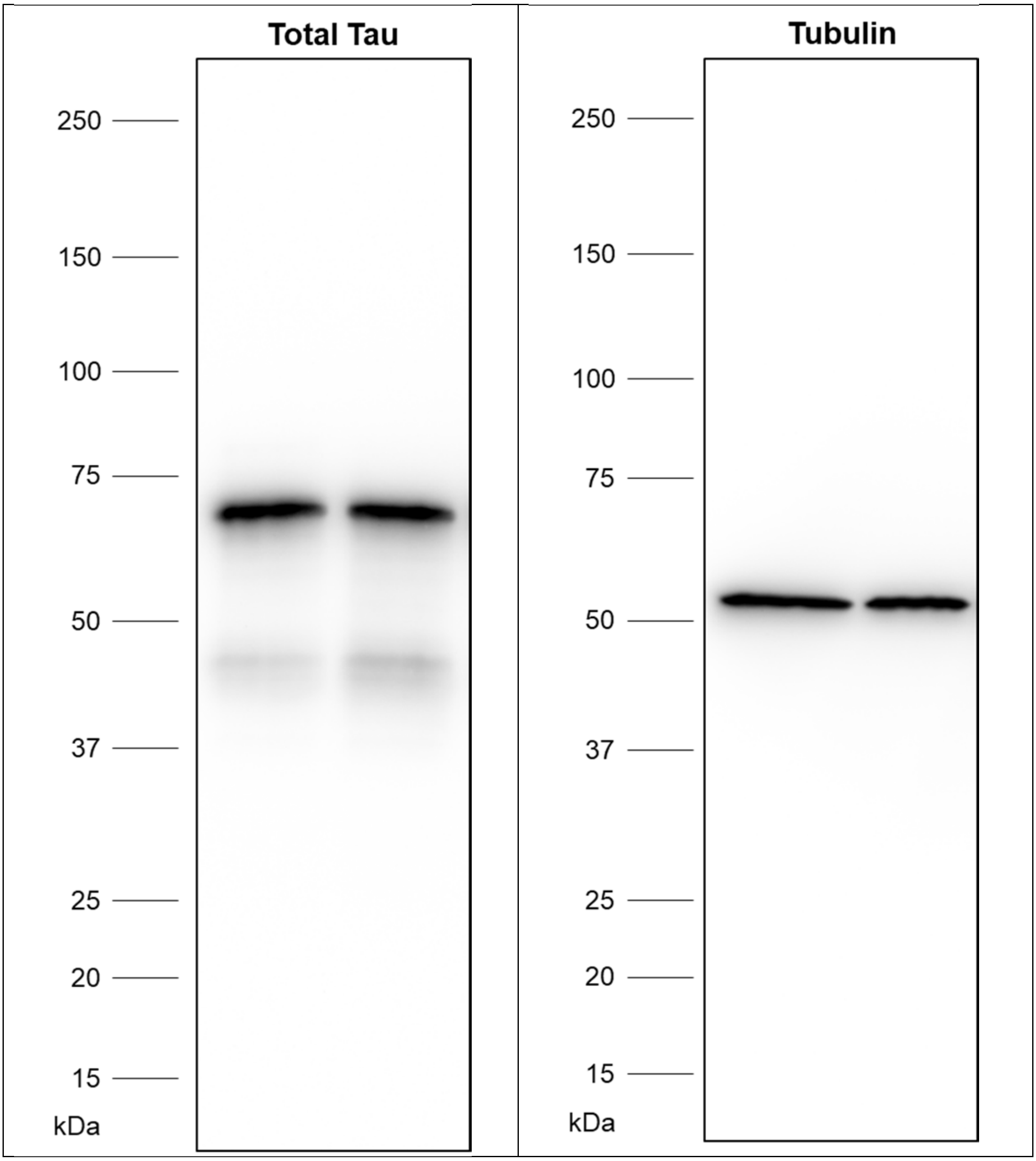

Figure 2b: *xbp-1* loss of function in a Tau (low) B background increases soluble tau protein levels. Representative full-length immunoblots of total tau and tubulin are shown for one of three independent biological replicates for each genotype. Loading order from left to right in each blot is Tau (low) B, Tau (low) B; *xbp-1* (-/-).

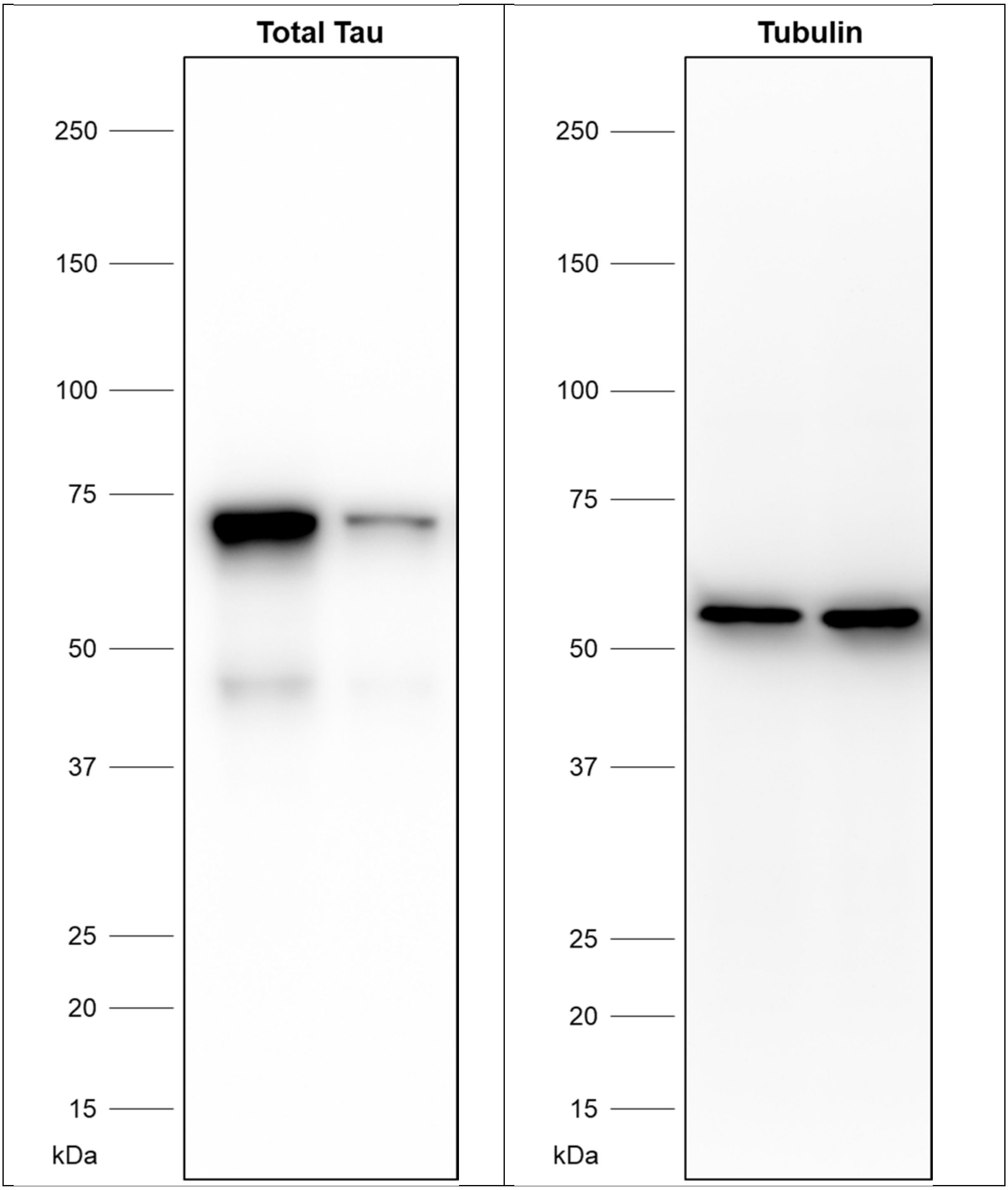

Figure 3b: Neuronal overexpression of *xbp-1*s in Tau (high) background decreases different soluble tau protein species. Total tau protein levels are decreased in Tau (high); *xbp-1s* Tg animals. Representative full-length immunoblots of total tau and tubulin are shown for one of three independent biological replicates for each genotype. Loading order from left to right in each blot is Tau (high), Tau (high); *xbp-1s* Tg.

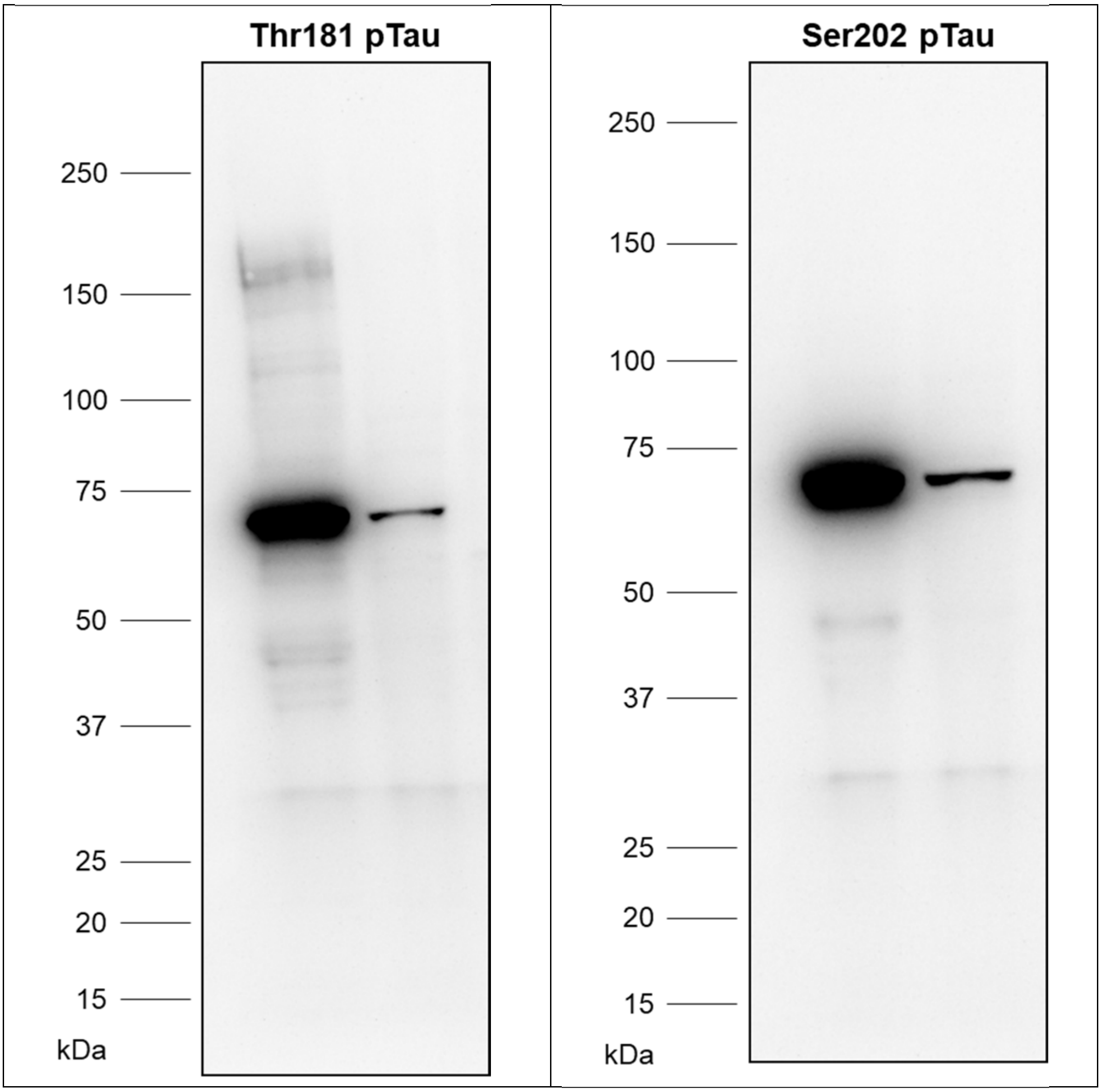

Thr181 pTau
250
150
100
75
50
37
25
20
15
kDa
Ser202 pTau
250
150
100
75
50
37
25
20
15
kDa

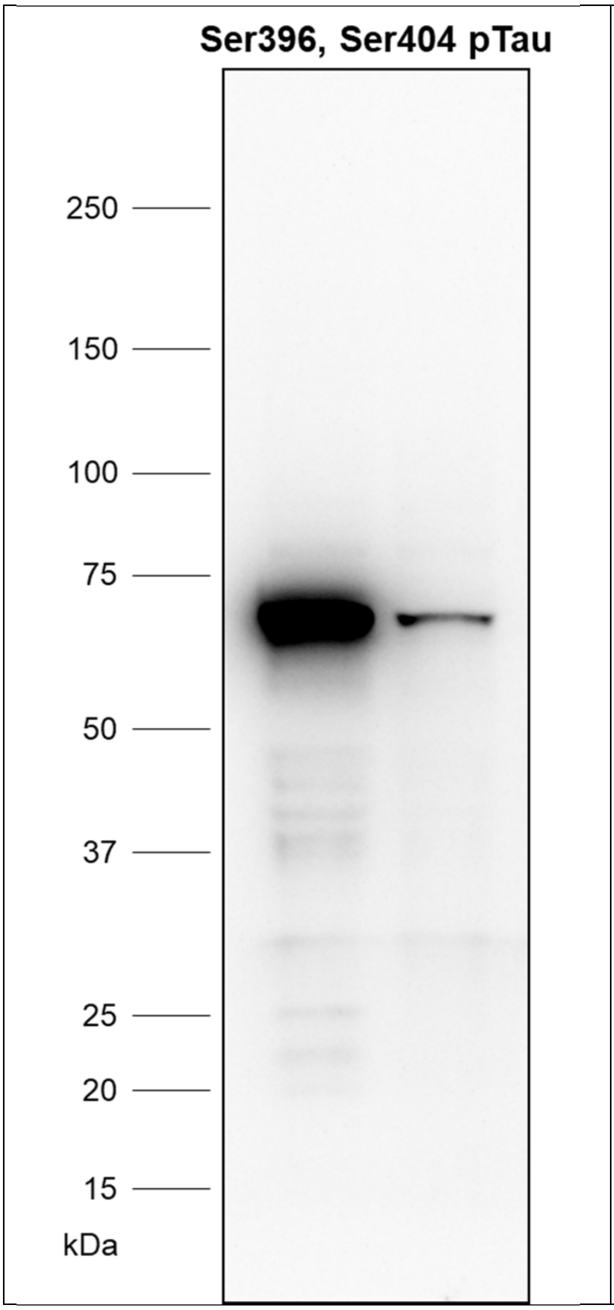
Ser396, Ser404 pTau
250
150
100
75
50
37
25
20
15
kDa

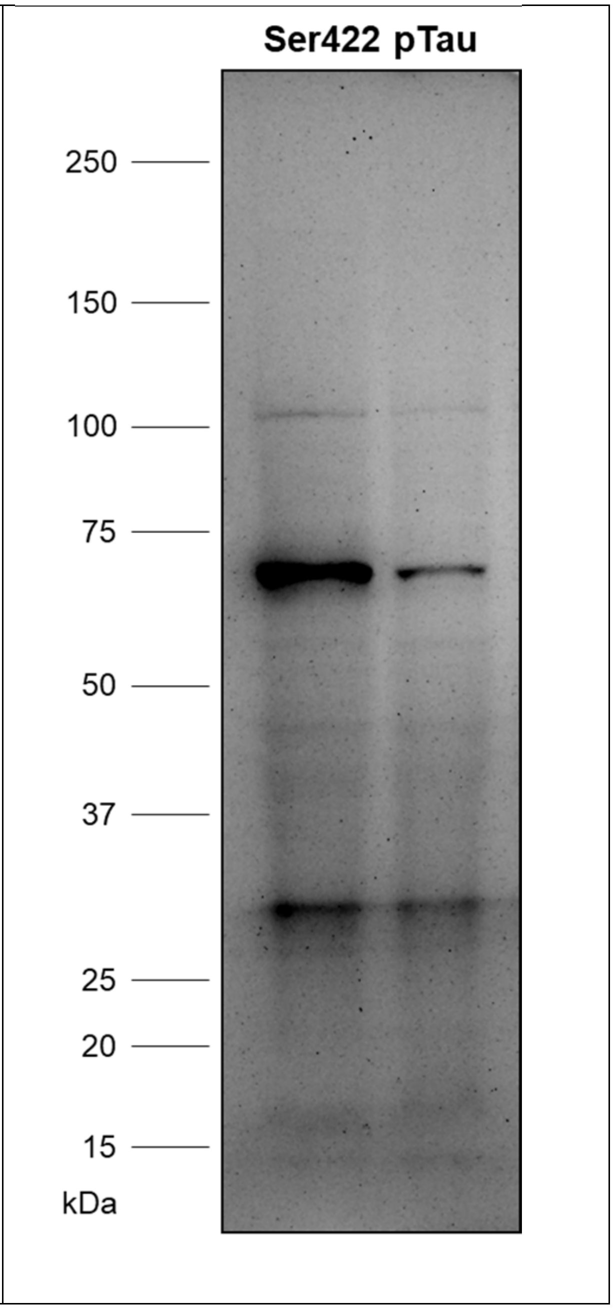
Ser422 pTau
250
150
100
75
50
37
25
20
15
kDa

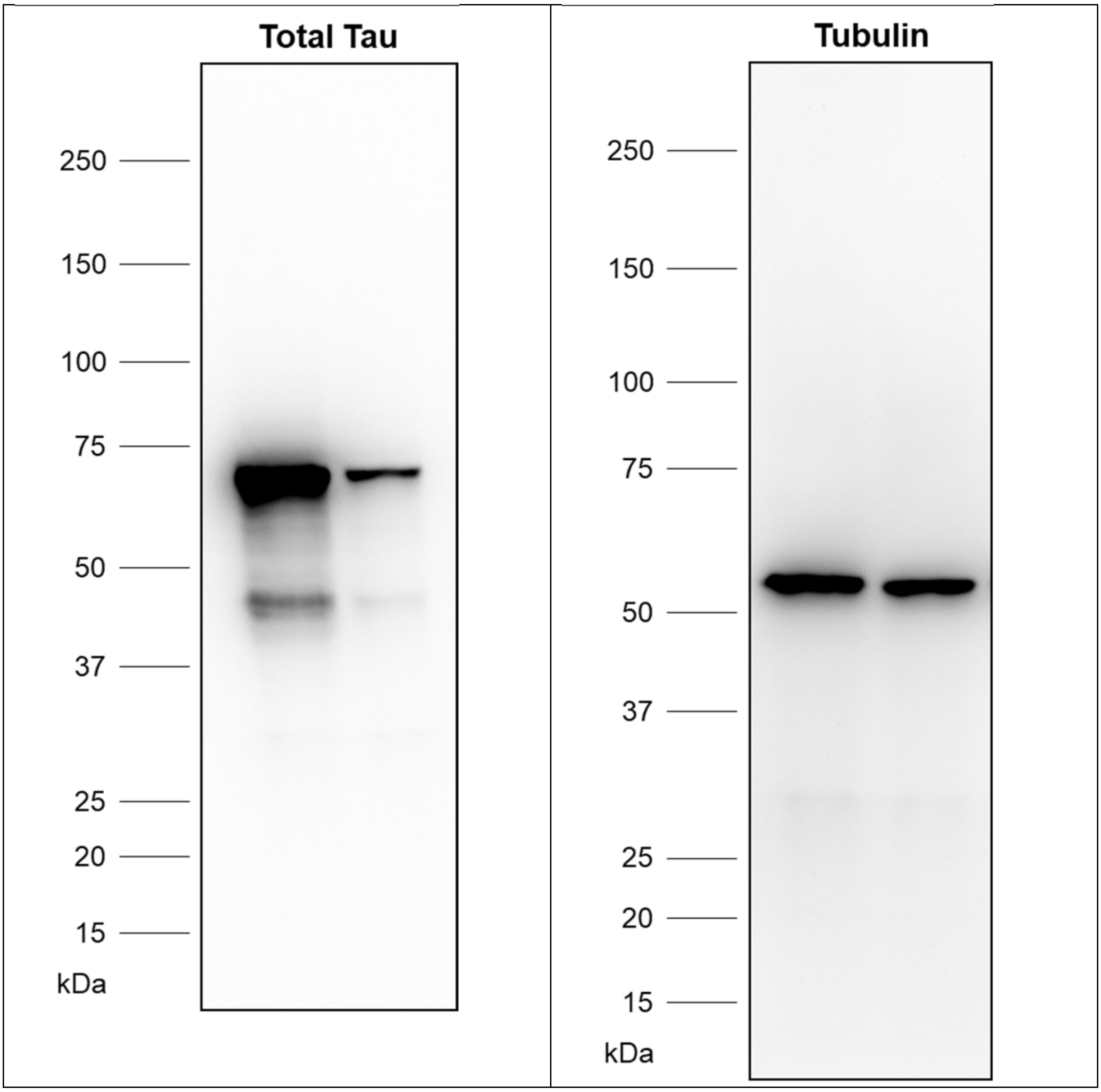

Figure 3d: Neuronal overexpression of *xbp-1*s in Tau (high) background decreases different soluble tau protein species. Phosphorylated and total tau protein levels are decreased in Tau (high); *xbp-1s* Tg animals. Representative full-length immunoblots of phosphorylated tau at Threonine 181, Serine 202, Serine 396/Serine 404, and Serine 422, total tau, and tubulin are shown for one of four independent biological replicates for each genotype. Loading order from left to right in each blot is Tau (high), Tau (high); *xbp-1s* Tg.

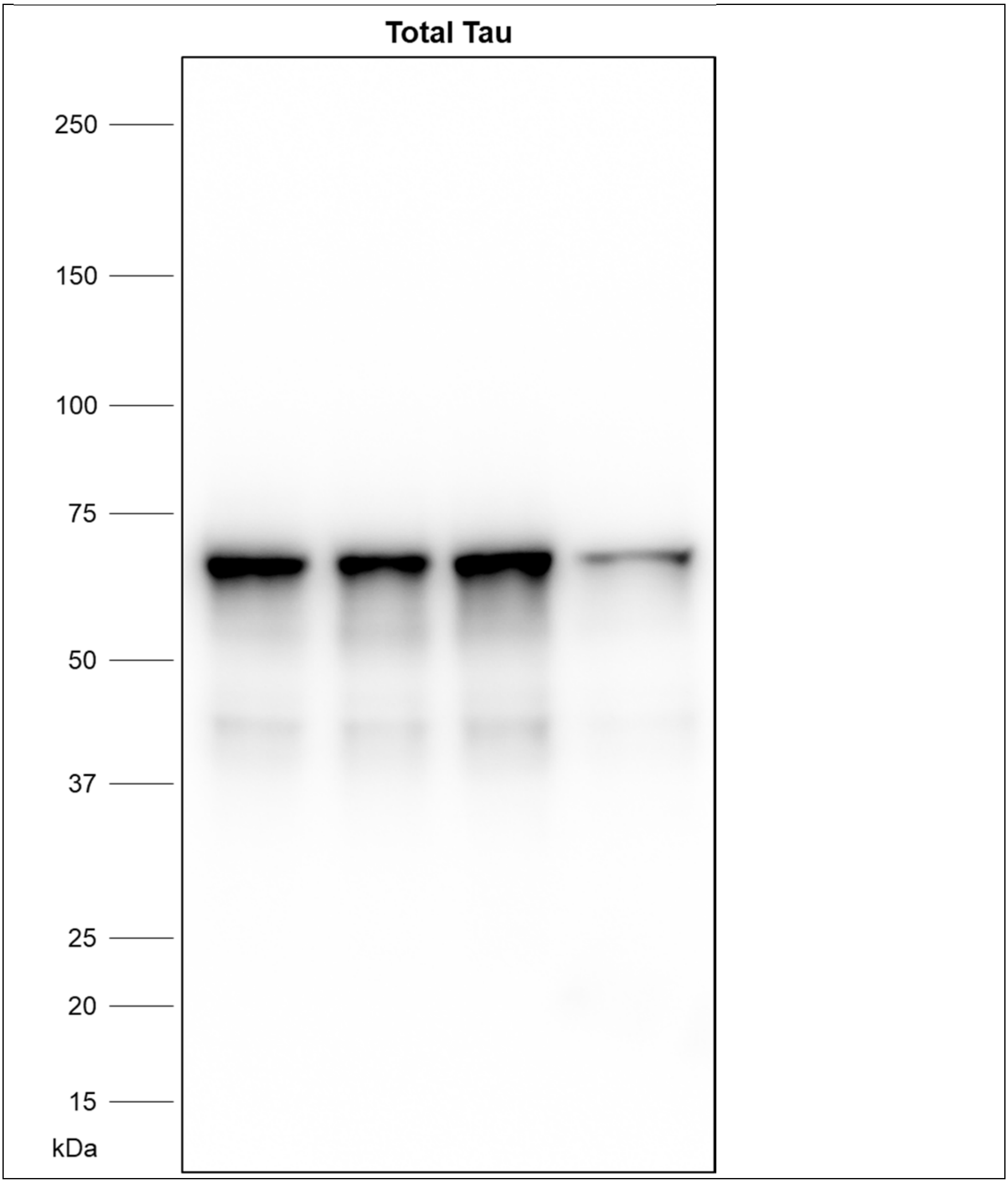
Total Tau
250
150
100
75
50
37
25
20
15
kDa

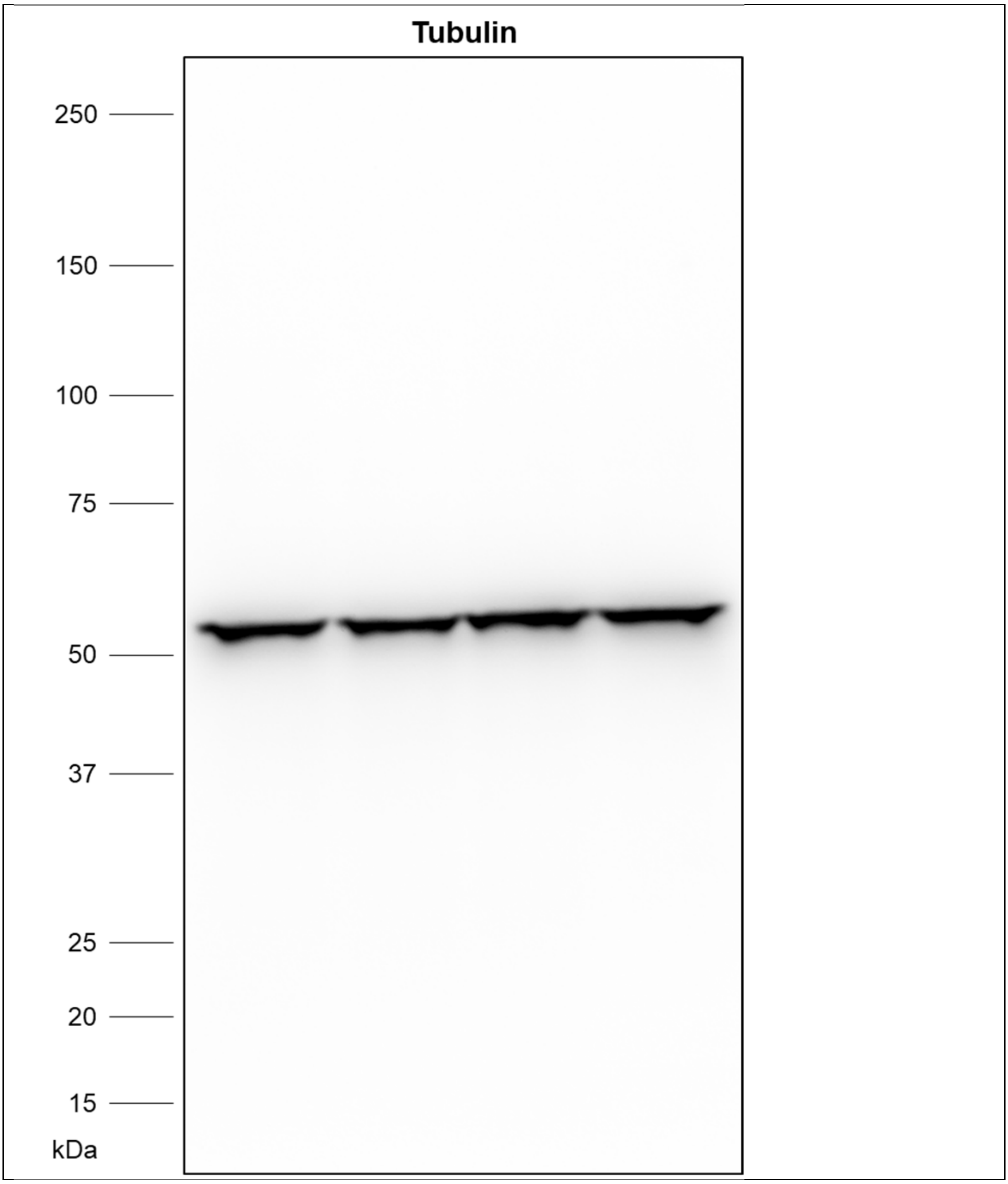

Figure 4c: *atf-6* (-/-) B prevents *xbp-1*s Tg-mediated decreases in total tau protein levels in Tau (high). Representative full-length immunoblots of total tau and tubulin are shown for one of four independent biological replicates for each genotype. Loading order from left to right in each blot is Tau (high), Tau (high); *atf-6* (-/-) B, Tau (high); *xbp-1s* Tg; *atf-6* (-/-) B, Tau (high); *xbp-1s* Tg.

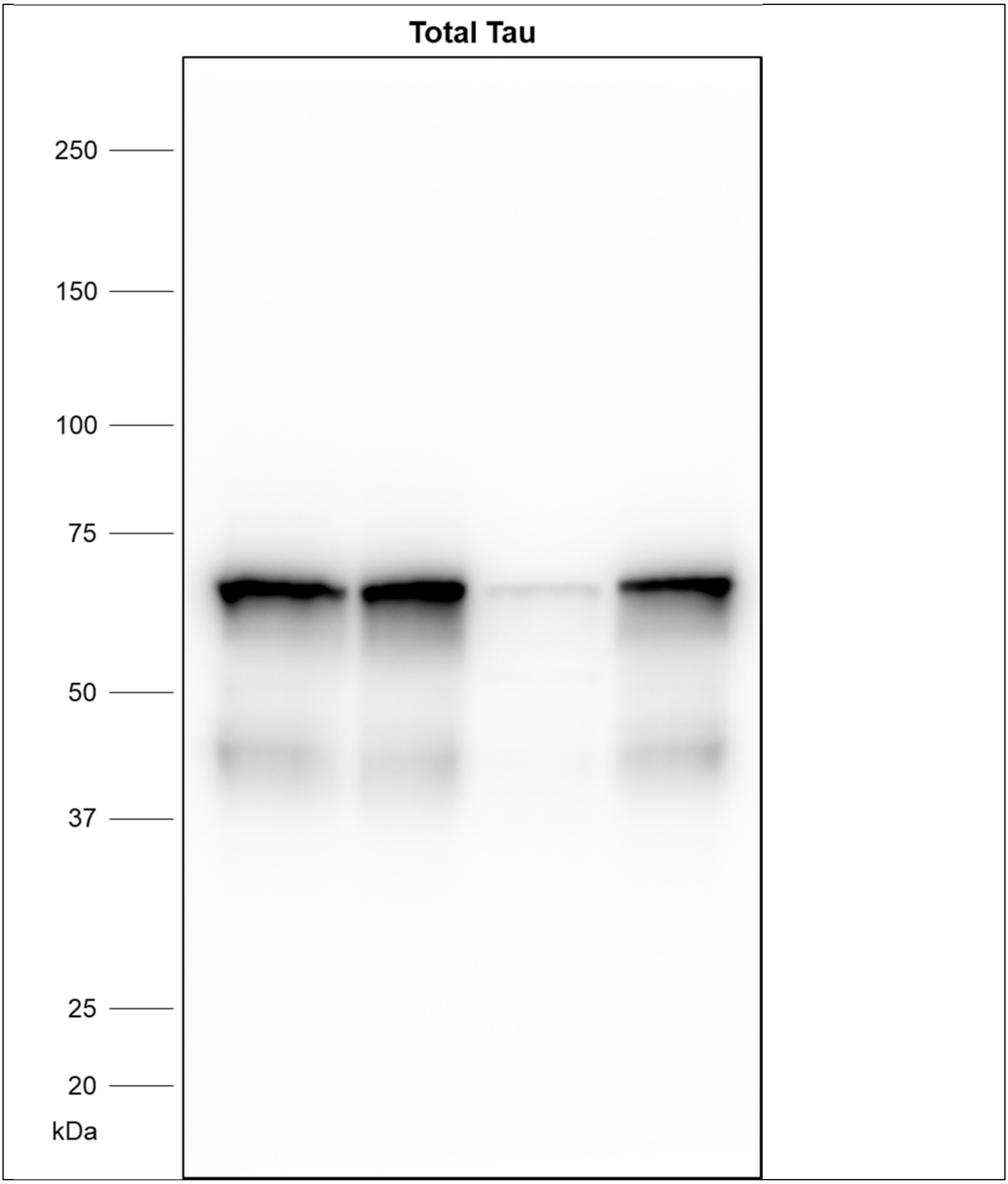
Total Tau
250
150
100
75
50
37
25
20
kDa

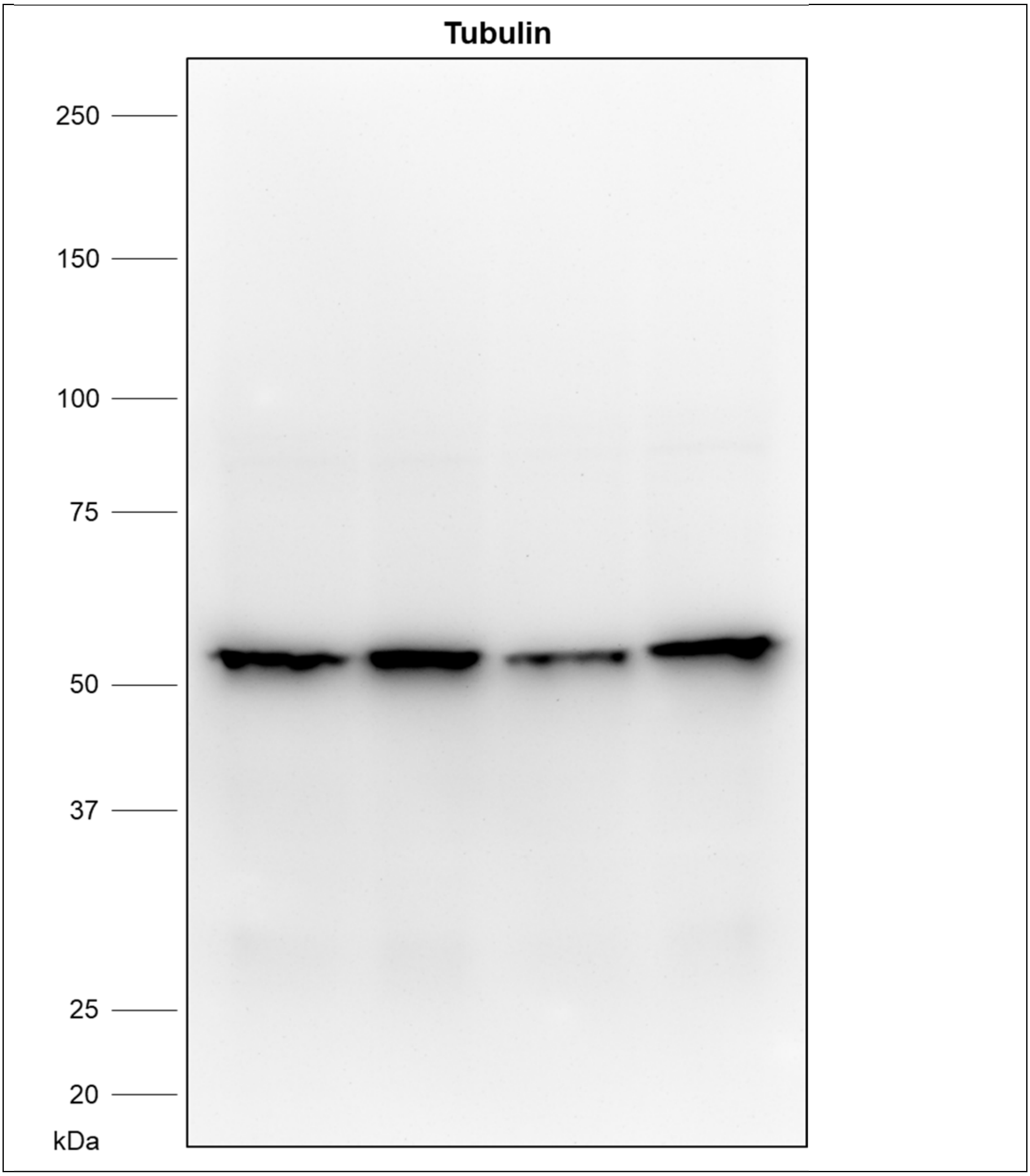

Supplementary Figure 7b: *atf-6* (-/-) A reverses the ability of *xbp-1s* Tg to decrease total tau protein levels in a Tau (high) background. Full-length immunoblots of total tau and tubulin are shown for one independent biological replicate for each genotype. Loading order from left to right in each blot is Tau (high), Tau (high); *atf-6* (-/-) A, Tau (high); *xbp-1s* Tg, Tau (high); *xbp-1s* Tg; *atf-6* (-/-) A.
